# Crohn’s Disease in Malaysia: Could Application of the Precautionary Principle Reduce Future Incidence?

**DOI:** 10.3390/microorganisms14020295

**Published:** 2026-01-27

**Authors:** Roger W. Pickup, Pei Boon Ooi, Gaurav Agrawal, Peter M. Atkinson, Jeremy Sanderson, Raja Affendi Raja Ali

**Affiliations:** 1 Division of Biomedical and Life Sciences, Faculty of Health and Medicine, Lancaster University, Lancaster LA1 4YW, UK; 2 Faculty of Medical and Life Sciences, Sunway University, Bandar Sunway, Petaling Jaya 46150, Selangor, Malaysia; peiboono@sunway.edu.my (P.B.O.);; 3 Department of Nutritional Sciences, School of Life Course & Population Sciences, Faculty of Life Sciences & Medicine, Kings College, London SE1 9NH, UK; gaurav.agrawal@kcl.ac.uk (G.A.); jeremy.sanderson@gstt.nhs.uk (J.S.); 4 Lancaster Environment Centre, Faculty of Science and Technology, Lancaster University, Lancaster LA1 4YW, UK; 5 IBD Centre, Guy’s and St Thomas’ NHS Trust, London SE1 7EH, UK

**Keywords:** inflammatory bowel disease, Crohn’s disease, ulcerative colitis, *Mycobacterium avium* subspecies *paratuberculosis*, Malaysia, disease control, surveillance, human health

## Abstract

Inflammatory bowel disease (IBD) comprises mainly Crohn’s disease (CD) and Ulcerative Colitis (UC). The Western model suggests that environmental factors, immunological factors, the gut microbiome, and genetic disposition all contribute to the onset and sustained symptoms that define CD, although the pathogenesis of CD remains unresolved. Current studies propose that in individuals who are genetically susceptible, genetic factors linked to immune dysregulation, in combination with environmental exposure, can result in dysbiosis of the gut microbiome and intestinal barrier dysfunction, leading to immune dysregulation. In Malaysia, the incidence of IBD is rising with CD increasing disproportionally compared to UC, and the incidence of CD currently mirrors that of the United Kingdom in the 1930s, which now has one of the highest incidences worldwide. Given the suggested role of *Mycobacterium avium* subspecies *paratuberculosis* (MAP) in CD in Western countries, which is subject to some controversy, this review summarises for the first time the current evidence on genetic, environmental, and microbial factors that could contribute to the rise of Crohn’s disease in Malaysia and proposes preventive approaches. We note the increasing reliance of Malaysia on imported cattle and milk products from areas of high Johne’s Disease prevalence to meet increasing demand and changes in milk preferences in the Malaysian population, both key indicators for human-MAP exposure in the Western model. Therefore, should MAP be shown to be associated with CD in Malaysia, some preventative measures are suggested, such as screening imported and native beef and dairy cattle, dairy products and ultimately water, both recreational and potable.

## 1. Introduction

This review focuses on Crohn’s disease (CD) in Malaysia including increasing trends in its incidence, increasing trends in cattle imports and consumer demand for dairy products which, in the future, may link to an association with JD in Malaysia, and uses the experience of the UK historically to make some recommendations for possible mitigations in Malaysia.

Inflammatory bowel disease (IBD) encompasses conditions such as CD, Ulcerative Colitis (UC), Microscopic Colitis and Indeterminate Colitis, all of which are characterised by chronic inflammation of the gastrointestinal (GI) tract [1,2,3]. IBD is a chronic, multifactorial GI disease with various manifestations across the world [4]. It is a persistent inflammatory illness of the gastrointestinal tract (GIT) triggered by an inappropriate immune response to environmental stimuli in genetically predisposed persons, a feature common to East and West. It is typically a disease of early adulthood with the initial onset of symptoms in patients usually in their early 20s [5]. It is estimated that approximately 7 million people worldwide are affected by IBD, and that this number is increasing [6,7,8]. The disease is considered specific to industrially developed Western countries, with the highest rates detected in North America and Europe [9,10,11,12], and with the UK having one of the highest rates worldwide [13]. 

The incidence of IBD has increased in newly industrialised regions such as the Middle East, Asia and South America [13]. While industrialization and the incidence of IBD have increased in these regions since the late 20th century, regional disparities exist, as shown for Asia across its large population of approximately 4.6 billion in 2020 (Table 1). This spatial variation in IBD incidence or prevalence may reflect differences in distribution, diagnosis and recording. Despite this, one constant has been that the incidence of UC is greater than that of CD [14].

Recently, global development of IBD has been shown to occur in evolutionary stages which differ depending on the IBD status [15]. Western countries are classified as stage 2 (acceleration in incidence), 3 (compounding prevalence; the incidence decelerates, plateaus or declines while the prevalence steadily increases) or 4 (Prevalence equilibrium where prevalence plateaus due to shifts in an ageing IBD population). In contrast, Malaysia, the focus of this comparative review, is classified as stage 1 (emergence) [15]. 

Malaysia is now confounding the UC > CD trend. In 2015, the approximate incidence rate of Crohn’s disease was 0.20 per 100,000 persons with the highest among the Indian population [16]. During the period 2010 to 2018, CD incidence rose significantly. This was exemplified by a reducing UC:CD ratio: 5:1 (1980–1999), 8.1 (1990–2000), 3.6:1, 1.9:1 (2000–2009) and 0.77:1 (2010–2018) [17,18]. For Malaysia, as with most of Asia, the trends in IBD have an unclear aetiology, but are perhaps beginning to mirror historical ‘western IBD’ trends. Chew et al. (2023) suggested that a better understanding of the epidemiology and of the exposomes will lead to better health care planning [17]. Therefore, the role of environmental factors represents an area for further study, and the role of *Mycobacterium avium* subspecies *paratuberculosis*, as part of the exposome in the western model, may have some relevance to Malaysia, with its current rising CD incidence and changing lifestyle patterns [19]. 

This is the first review to focus on the increase in IBD in Malaysia [20] with specific reference to Crohn’s disease and the potential role of *Mycobacterium avium* subspecies *paratuberculosis* (MAP), an organism that is implicated in CD pathogenesis. The review synthesises the genetic, environmental, and zoonotic factors potentially influencing CD in Malaysia and proposes future disease mitigation strategies [17]. 

## 2. Epidemiology and Crohn’s Disease in Malaysia

The incidence/prevalence rates of CD (and IBD in general) are currently low in Malaysia, but the incidence is increasing and increasing relative to UC, and marked racial differences exist [18]. In comparison, CD incidence in the UK per 100,000/year in the 1930s was 0.18, rising to 5.95 in the 1970s, and is now 10.2 [5,21]. Malaysia lags behind the UK/Europe/USA/Australasia [17], but the rapid increases are apparent. 

Crohn’s disease (CD) is a chronic, relapsing and remitting transmural inflammation that can affect almost any site along the entire gastrointestinal tract. Chronic inflammation in CD is characterised by progressive fibrosis with subsequent strictures and deep penetrating ulceration, leading to abscess or fistula formation [22]. The location of inflammation in the mucosa of the intestine, most commonly in the lower part of the small intestine, can cause inflammation anywhere in the gastrointestinal tract, from the mouth to the anus. The most common areas of involvement are the distal small bowel and colon, especially the terminal ileum and caecum [22]. Unlike CD, ulcerative colitis (UC) affects the mucosal layer of the colon, causing lesions in the large intestine and rectum. CD and UC can usually be distinguished histopathologically with characteristic features unique to each [23].

The pathogenesis of CD remains unclear; it is suggested that environmental factors [20,21,22,23], immune factors [24], gut microbiome [24] and genetic disposition [25] all contribute to the onset and sustained symptoms that define CD. Environmental exposures for genetically susceptible individuals can cause dysbiosis of the microbiota of the gut and dysfunction of the intestinal barrier, subsequently interacting with immune dysregulation [26]. MAP, therefore, which may colonise an individual’s gut, is not removed by the immune response, but is merely contained and survives in macrophages and other reticuloendothelial cells [25]. Although the exact mechanism remains unclear, a “triggering event” activates this latent infection, leading to CD onset [27,28]. 

Recently, IBD (CD and UC) has increased to become a global disease, [15] with IBD incidence rising rapidly in the East while plateauing in the West [29]. Moreover, the course of IBD differs between East and West, as does its clinical presentation, as shown in Table 2 [19].

This is reflected in CD in the East, presenting as a more complex disease such as with a more penetrating/stricturing phenotype and more perianal and upper gastrointestinal (GI) tract involvement [29]. The variations in IBD incidence/prevalence may be due to differences in the population distribution, and there are great regional disparities in Asia, given its large, diverse population [14]. In 2024, the Malaysian population comprised Malays and Bumiputeras (69.9%), Chinese (22.8%) and Indians 6.6% [30].

The specific characteristics of IBD in Southeast Asia (SEA) are similar to those in East Asia and the West [19] In contrast with the West, risk factors such as familial aggregation and smoking are not significant in SEA patients with CD [17,31]. In multiracial populations, such as those in Singapore and Malaysia, Indians have the highest incidence and prevalence rates, which is likely due to important mutations, including the NOD2 predisposing mutation SNP5 and IBD risk alleles IGR2198a and IGR2092a [17].

The effects of genetic and environmental risk factors contributing to IBD also differ between Eastern and Western populations. Considering the differential effects of genetic and environmental risk factors in the East and West, consideration needs to be given to future risks to mitigate and prevent further dramatic increases [14,17,19]. In Malaysia, in 2015, the approximate incidence rate of Crohn’s disease was 0.20 per 100,000 persons with the highest incidence among the Indian population, although susceptibility is found in the Malay and Chinese ethnic groups [16]. 

The incidence/prevalence rates of CD (and IBD in general) are currently low in Malaysia, but the incidence is increasing and increasing relative to UC, [18]. The rapid increases seen require mitigation to prevent the continuing upward trend of CD (and IBD) from rising further, with consequential impacts on the patient, health care costs and the economy. This mitigation may be required now to stem the increase and would potentially involve actions not taken in the West when the incidence was at this low level. The epidemiology of CD, as influenced by genetic susceptibility and the current trends in CD, offers an opportunity to identify the environmental exposure routes that may also contribute to the increasing disease burden [17]. 

## 3. Genetic Susceptibility and Ethnic Variation

CD is influenced by ethnicity (for Malaysia, the Chinese, Indian, and Malay are the dominant groups), age, familial aggregation, and genetic predisposition [17]. More than 200 risk genes have been identified for IBD [32,33]. Many of the major genes are related to CD, most notably, mutations in *NOD2*, *ATG16L1* and *IRGM*, suggesting a role for altered bacterial interactions, particularly autophagy, in disease pathogenesis. Gene mutations in both the innate (*CARD9*, *IL23R*, *STAT3*, *TLR4*) and adaptive (*HLA*, *IRF5*, *PTPN22*, *TNFSF15*) immune pathways may also be involved [32]. CD and other immune-related diseases have some genetic overlap with approximately 30% of associated variants in these initial studies being shared with UC. Immune-mediated diseases, for example, coeliac disease, rheumatoid arthritis, and type 1 diabetes, share around 50% of loci, showing the interrelated genetic complexity of these diseases [34].

Despite genome-wide association studies for IBD being performed mainly in Caucasian populations, Chew et al (2023) reviewed the genetic mutations that impact the Southeast Asian population [17], most notably polymorphisms in *NOD2*, *ATG16L1*, *ATG16L2*, *LINC00824*, *IBD5*, *IRF5*, *CXC16*, *TLR4*, *DLG5 JAK2* as described in Table 3. 

IBD in diverse Asian regions carries a unique disease phenotype including male predominance, increased perianal complications and high frequency of perianal fistula in CD [46]. Moreover, this phenotype was reflected in Asian immigrants in North America and Europe, with Asian Europeans suffering more perianal complications [46]. Indians in multiracial populations, such as in Singapore and Malaysia, have the highest incidence and prevalence rates. This population carried a higher frequency of the *NOD2* predisposing mutation, SNP5, and the IBD risk alleles IGR2198a and IGR2092a [17]. 

Given the above for CD, there is no ‘one size fits all’ due to the multiracial makeup of the Malaysian population, this highlights that more studies are needed to identify characteristics of the multiracial Asian population to improve clinical outcomes [47].

## 4. Environmental and Microbial Factors

**Figure 1:**
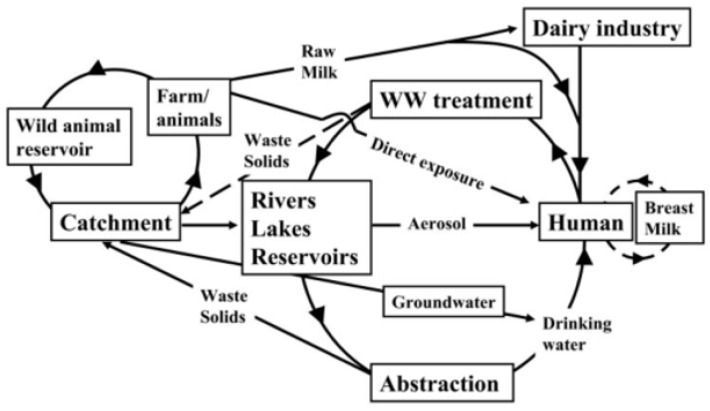
Model of sources and sinks of *M. avium* subsp. *paratuberculosis* in the environment with respect to human exposure (adapted from [61]).

Johne’s Disease (JD) is a contagious disease that can infect ruminant and non-ruminant species, including primates [48,49,50] with a significant economic impact on cattle and sheep farming. JD is endemic worldwide with a high herd prevalence in the UK, USA, Europe, India and Australasia [51]. *Mycobacterium avium* subspecies *paratuberculosis (MAP)* is the etiologic agent of JD in animals, causing a chronic and progressive intestinal disease [52]. MAP is an obligate pathogenic bacterium and a slow-growing member of the genus *Mycobacterium*. It has a remarkable capacity to persist and survive in the animal host and in the environment [53]. JD, caused by MAP, has a huge worldwide impact on both economics and animal welfare. Given MAP’s significant association with CD, it is now attracting public health concern [52,54]. Cows infected with MAP excrete the organism in their faeces and in milk within the udder. MAP may also enter raw milk through faecal contamination during milking [55]. Millar et al. [56] showed that MAP was present in retail milk, and this was confirmed by other studies in the UK and in other countries including Argentina, Brazil, Eire, India, Italy, the United States, the Czech Republic, and India, which reported MAP in a wide range of milk products [57], meat, and drinking water [58]. Due to possible human exposure, Kuenstner (2006) suggested that exposure to MAP was a public health issue [59]. Pickup et al. (2006) proposed a wider route for human exposure (Figure 1), in addition to dairy products, via the shedding of MAP from clinically and sub-clinically infected animals on pastures, catchment run-off driven by rainfall, and exposure via aerosols from domestic water (including domestic showers) and rivers [60,61]. The latter is suggested to explain an observed increase in CD incidence near water bodies, where long-term exposure to MAP increases the risk of developing CD in susceptible individuals, as shown by CD clusters significantly associated with the River Taff in Cardiff, UK [60,61,62]. 

With respect to pathogenicity, some MAP antigens closely resemble human tissue antigens causing an autoimmune response via molecular mimicry enhanced by MAP’s ability to persist within the host’s macrophages. This can lead to altered antigen presentation and T-cell activation [63], and a failure to distinguish between MAP/human antigens, resulting in chronic inflammation and autoimmune conditions. Examples of such diseases, in addition to CD [64] include Hashimoto’s thyroiditis [65], Type 1 diabetes [65], sarcoidosis [65], multiple sclerosis [66] and rheumatoid arthritis [67].

## 5. Lifestyle and Dietary Influences

Western dietary patterns high in refined grains, red and processed meats, saturated fats, animal fats, and low in fruits and vegetables have been consistently associated with an increased risk of developing IBD [68]. However, screening in the UK found that 92% of CD patients, compared 26% of non-CD patients (non-inflammatory bowel disease controls, as well as ulcerative colitis patients), carried MAP in fresh mucosal biopsy samples [69,70]. However, not all laboratories could confirm this, so by combining both positive and negative results from laboratories worldwide, Feller et al. [71] showed that MAP was significantly associated with CD, but not, as yet, deemed causal. Alternative theories abound suggesting that MAP is a bystander organism [72] and others, such as Adherent *E. coli*, are implicated [73]. However, many studies showed that anti-mycobacterial antibiotic therapy induces remission in patients with active CD (e.g.， [74]) and more common features between JD and CD are being revealed with MAP as a common pathogen [75].

A correlation exists between the rising worldwide incidence of CD and the increased incidence of JD in dairy cattle over the last 100 years [12]. Agricultural areas report higher incidences of Crohn’s disease, although livestock workers do not experience an increased risk, despite working with infected animals; this may be a result of early protective exposure [28,64]. Where MAP of animal origin is at high levels in the environment, increases in CD exist such as in Winnipeg, Canada (3.5× increase over nearby areas; [76]), Cardiff, Wales [77,78] and Canterbury, New Zealand, as described by McNees et al. [79]. 

The importation of MAP-infected animals has been documented to precede an increase in CD incidence [40]. In 1933, an epidemic of JD in Iceland caused by the importation of infected Karakul sheep was followed by a JD epidemic in cattle. The CD incidence rates then rose 18-fold between 1960 and 1992 [80]. Similarly, in the Czech Republic, a 4.5-fold increase in CD was reported between 1995–2007, after the importation of asymptomatic infected cattle, as noted by Hermon-Taylor in 2009 [49].

Japan has seen an increase in CD where prevalence has increased substantially from 34.2 in 2010 to 54.5 per 100,000 population in 2019. This increase was noted in both males and females aged between 6 and <65 years [81]. However, the incidence was almost fourfold lower than in the equivalent populations in the USA [81]. It was suggested that the increased westernisation of Japan’s diet has had a significant influence [82], with dairy products rising from 57.4 g per capita/day to 127 g per capita/day and, thus, an increased dietary intake of animal protein and n-6-polyunsaturated fatty acids, with a decreased intake of n-3 polyunsaturated fatty acids. This is a possible factor in the development of CD [83], although Kodama et al. (2024) suggested that an increase in meat and a decrease in fruit consumption had a role [84]. Therefore, changes in lifestyle, particularly towards a western diet or migration from East to West, can increase CD risk [15].

Possible routes for human exposure to MAP have been described previously and include both environmental and lifestyle components linked to JD in animals, particularly cattle and sheep [63,64,65]. The emergence and increase in CD generally follow the modified Hruska postulate of Crohn’s disease, which states that an increase in CD is preceded by the importation of animals with JD and that milk from imported diseased dairy animals exposes the population to MAP. Moreover, increased use of baby formula may reduce immune resistance to infection in the infant population (see Section 5.3) [85,86].

### 5.1. Increase in Beef Demand: Johne’s Disease in Malaysia

In 2022, the cattle stock in Malaysia was approximately 720,000 head of cattle. This indicates an increase compared to 2020, although over the period 2012–2022 it has declined [87]. Importation of cattle to Malaysia is needed to meet the growing demand for beef and milk to supplement local production [87]. Importation is overseen by the Malaysian Department of Veterinary Services (DVS). With cattle, among other diseases, comes the threat of JD, which is low in Malaysia: between 2001 and 2018, only two positive cases of bovine JD were recorded in 2001 and 2018, respectively [88], although others were suspected in cattle and buffalo [89,90]. In Malaysia, the DVS has formulated several disease surveillance, control, monitoring, and eradication programs/protocols of livestock/zoonotic diseases, including for JD [91]. This is extremely important given the endemic nature of JD worldwide [92] and the current importation of cattle from countries identified as endemic for JD.

The ruminant industry in Malaysia has become dominated by importation, where the value of imports for live cattle has risen, from Malaysian ringgit (MYR) 82.8 million in 2022 to MYR 108.7 million in 2023, to satisfy domestic consumption, against a background of a worldwide epidemic of JD [51,93]. However, the domestic supply of beef and buffalo has declined. As a result, the self-sufficiency rate for beef and buffalo fell from 23.6% in 2015 to 14.7% in 2022. Consequently, 85.3% of the beef and buffalo meat supply in Malaysia must be imported. In 2022, the top partner countries from which Malaysia imports animals include India, New Zealand, Australia, Thailand, and China. Of these, India, New Zealand, and Australia are each noted for their high JD incidence [94,95].

### 5.2. Milk Production and Consumption in Malaysia: Increased and Changing Demand

Expansion of MAP within domestic milk-producing animals precedes the appearance of CD [86], as detailed previously for Iceland and the Czech Republic. Furthermore, in 2007, the US Department of Agriculture (USDA) acknowledged that 70% of US dairy herds contained MAP-infected animals [96]. In Japan, 54% of MAP-infected animals detected by the Japanese Quarantine Service came from the United States [97]. China, an exporter of cattle to Malaysia, has reported a 2-12% incidence of MAP-infected cattle in different regions [98]. Importation of clinically and sub-clinically infected animals will make the Malaysian herd stock vulnerable to JD and, therefore, increase the potential for exposure of the population to MAP.

**Figure 2:**
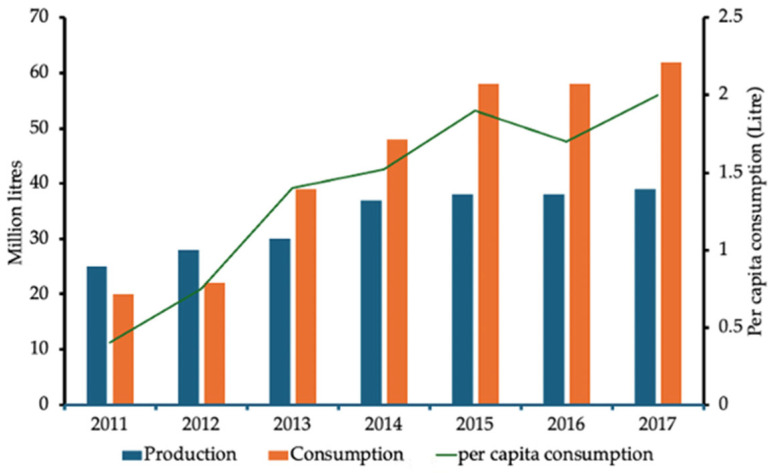
National milk production and consumption, and per capita consumption in Malaysia (adapted from [99]).

Due to limited domestic production of dairy caused by relatively low numbers of dairy cattle, Malaysia relies heavily on imported dairy products, including milk and cheese. Milk production in Malaysia has decreased over the last decade, with a resulting increase in importation as supply tries to meet demand (Figure 2), which itself has increased. Now Malaysia depends on imports to meet demand for dairy products. For example, importation of dairy products increased by 6% between 2018 and 2022, resulting in a decline in milk self-sufficiency from 76.6% to 59.02% (2019), with Malaysia importing over 458,000 tones of dairy products, worth USD $1.5 billion in 2022. 

Suntharalingam (2019) reported that in 2011, an average Malaysian consumed 0.4 litres of milk in a year; at the end of the following 6 years, the consumption per capita increased to 2 litres/year (Figure 2) [99]. Increasing salaries and more awareness of the nutritional benefits, coupled with a change in taste preference, have driven an increasing preference for milk and dairy products [100]. Data from Suntharalingam (2019) showed that since 2013, demand has outstripped supply, hence the need for imports and the lowering of self-sufficiency and that the market is expected to continue to grow [99].

In 2024, Malaysians consumed approximately 80 million litres of milk, with an increase in consumption averaging 8% since 2010, with half of the milk imported. Milk imports have increased from 1.8 million tonnes to 2.3 million tonnes from 2012–2014 to 2021–2022 [101]. To sustain this growth, at least another 30,000 cows are needed, as children are encouraged to drink fresh milk in schools. Without this, Malaysia will rely on supplementary milk importation. Malaysian consumers’ dietary preferences are also changing. In 2022, the Malaysian market comprises ultra-high temperature treated milk (UHT) and pasteurised milk, as well as condensed milk, with 47% of consumers drinking only UHT and 33% drinking only pasteurised milk, with 20% drinking both types [102]. The trend for drinking pasteurised milk is increasing instead of UHT and condensed milk (65%), where the risk of MAP exposure is less [101,103] 

In Malaysia, milk for many is not an option, as lactose intolerance is prevalent in the Asian communities. In Malaysia, the rate of lactose intolerance is extremely high amongst adults at 88% for Malays, 91% for Chinese, and 83% for Indians. Lactose malabsorption was found in 61.5% Malay and 54.5% Chinese children. Underlying this lactase non-persistence is a greater prevalence in those descended from East Asian populations [104]. 

### 5.3. Relating to Newborns

Breastfeeding confers protection against future development of CD which suggests that protection in the infant is lost with increasing use of baby and infant formula [105]. It is predicted that the use of infant formula milk will rise by 10% per annum from 25 million kg in 2018 to 33 million Kg in 2030 [101] coupled with the possible presence of MAP in infant formula, albeit detected only by molecular signal so far [105,106]. As a consequence of enhanced virulence of viruses and exposure to mycobacteria in milk in the immediate neonatal period, where the absence of acquired immunity results in exaggerated tissue destruction by viruses and mycobacteria [86] or a newborn lacking co-functioning acquired/adaptive immunity, the inherent immune response needed to terminate Mycobacterial replication is severely compromised [86]. Monif [107] expressed the view that the global epidemic can be reduced by women breastfeeding their newborns for the first four weeks of life or using an infant formula that is MAP-free. 

### 5.4. Water

Deposition of MAP from clinically and sub-clinically infected animals occurs on pasture that lies within river catchments [61]. In countries with JD, it has been reported that human exposure to MAP can occur through the public drinking water supply [60,61,108] and via domestic shower aerosols, in addition to aerosols from rivers [62]. Entry into the public water supply begins with rain-driven runoff from land that enters rivers and reservoirs. From there, water is abstracted before purification, from which MAP is not removed; thus, MAP enters the domestic supply in households including showers [62]. Protection of reservoirs in large catchments is difficult due to land use practices and scale, but not impossible as shown by *Cryptosporidium* management in Australia which enforced animal restriction zones around reservoirs [109]. Drinking water in Malaysia is predominantly sourced from rivers and, as such, is vulnerable to microbiological and chemical runoff [110]. In Malaysia, water quality standards are in place [111]. As with worldwide standards, MAP is not a priority indicator of contamination.

## 6. Public Health Implications and Control Strategies

The incidence of IBD in Malaysia is rising, with CD also rising as the UC: CD ratio decreases [17]. Malaysia is currently at a point similar to that of the UK in the 1930s, when CD prevalence and JD were less common. There were no surveillance strategies in place in the UK in the 1960/70s and CD incidence in the UK rose from 0.18/100,000/year in the 1930s to 5.95/100,000/year in the 1970s, then decreased slightly to 4.8 in the 1980s [77,78]. Despite an increase in awareness of JD and possible implications of MAP, it rose to 10.2 in 2018 [5]. Currently, 500,000 people in the UK live with IBD which equates to around 200,000 to 250,000 with CD. 

### 6.1. Recommendations for Malaysia

Reflecting on the experience of the UK and using this as a prevention strategy, precautionary principles may apply and, as such, four main recommendations (8.1–8.4) can be made for Malaysia:

#### 6.1.1. Genetics

Genetic risk factors for CD are complex, given the racial composition of Malaysia (Malay, Indian, Chinese and others). Thus, education on how to reduce the risk is likely to become paramount, for example, through charities and patient sites. Educational awareness programs could focus on raising awareness of modifiable lifestyle risk factors; for example, suggesting that individuals with a family history of CD, especially a first-degree relative [112], may choose to look to alternative pasteurised milk products. Susceptible individuals could opt to reduce further exposure to risk factors such as those noted by Chew et al. [17].

#### 6.1.2. Malaysian CD Patient Screening for MAP

There is no information on the MAP status of Malaysian CD patients; therefore, screening to establish baseline levels is required using samples as UC controls. The presence of MAP can be ascertained using IS*900* PCR with confirmatory *F57* PCR [62,69] or by visualisation by FISH staining [113]. 

#### 6.1.3. Cattle Surveillance Strategy

As cattle imports increase to meet consumer demand, maintaining and improving surveillance for diseases such as JD becomes paramount. Should JD establish in the Malaysian herd, JD management strategies currently operating in several affected countries are available as exemplars. Perhaps adopting these strategies in herd management and husbandry now may produce an effective firebreak against an inevitable influx of diseased animals, which is especially important as sub-clinically infected individuals will be included in imports. 

#### 6.1.4. Milk Imports

Milk production on UK farms is subject to Food Standards Agency regulations and inspection [114]. All retail milk in supermarkets and shops is pasteurised, although raw milk can be purchased from farm outlets, which are guided by separate regulations [115]. Pasteurised milk must meet the UK microbiological standard [115]. However, UK standards do not require monitoring of MAP despite its continued proven presence in retail products [55] and its perception as a public health problem [59]. Implementation of the screening of milk could be upheld by using the precautionary principle [116] whereby the threat is perceived, and action is needed whilst evidence is gathered (an exemplar is with variant CJD in the UK, [117]). Thus, monitoring data confirming the presence or not of those perceived as susceptible could allow them to make appropriate lifestyle choices whilst not affecting the rest of the population. Malaysia can learn from and leverage the UK’s model to prevent future health challenges by addressing MAP risk in several ways, primarily by forming an interministerial task force. In Malaysia, the Ministry of Health, DVS and JAKIM (Jabatan Kemajuan Islam Malaysia, Islamic authorities) could pilot MAP surveillance for important high-risk dairy herds, execute voluntary MAP-Free certification for the dairy industry, create public awareness programs for those at-risk individuals and request universities to perform local MAP prevalence studies.

From an educational point of view, those considered susceptible could opt for a dairy-free trial period to reduce exposure to MAP, which is present in milk from countries with endemic JD [118], and/or observe, record and assess changes in CD symptoms while ensuring sufficient intake of calcium and vitamins from other sources (almonds, leafy greens) instead of milk products.

#### 6.1.5. Water Quality Surveillance

As with milk, water quality in the UK does not require MAP surveillance despite MAP being known to be present. Surveillance could, however, inform susceptible populations and allow them to access alternative sources of water (e.g., bottled water, which is often sourced from groundwater and springs that are not exposed to environmental animal activities, and for which there is no evidence of contamination with MAP). Again, Malaysia can leverage on its existing water quality framework with an additional focus on MAP surveillance, including MAP testing in the Malaysian water standard and establish treatment plants in areas with high CD prevalence. 

### 6.2. Future Vaccination and Therapeutic Strategies

Achieving optimal outcomes in CD relies not just on the appropriate use of current medical and surgical therapies, but also on careful attention to wider aspects of management, including early diagnosis, prompt initial management, close monitoring of treatment response, and psychological and dietary support [119]. Although CD has a high incidence and its aetiology is unresolved, an alternative treatment replacing or supporting current therapies could be provided by an anti-MAP therapeutic vaccine. Many studies have shown that Adenovirus-based viral vectors are safe for human use, coupled with the associated prime-boost strategies to increase immune responses, leading Sanderson et al. [120] to report the potential of a new CD vaccine currently undergoing trial. In addition, the gut microbiome has been suggested as a target for intervention in IBD pathogenesis and therapy [121], as have MicroRNA signatures which have a role in the pathogenesis and are possible targets for therapy [122]. Moreover, recent work by Aljabri et al. (2025) compared biologic and biosimilar therapies in IBD and emphasized that optimized drug delivery systems may improve therapeutic targeting and minimize adverse systemic effect, an approach that could be particularly valuable in developing healthcare settings such as in Malaysia [123,124].

### 6.3. Cost Implications

The annual UK care costs for any patient with CD in 2015 were £6156, with £19,800 for remission patients and £10513 for those who relapse [125]. Comparatively, the annual US costs in 2019 were estimated at $23,000 [126] and the Malaysian costs in 2016 were $15,000–$22,000 per annum [127]. These costs in the private health care area are a burden on the individual, in addition to loss of earnings, whilst the costs to the state are at an economic level. The cost of illness for the USA was found to be $22M and $15M for patients with ulcerative colitis and Crohn’s disease, respectively [128]. If CD rises in Malaysia, similar economic burdens could be imposed.

Surveillance has a cost which initially could be considerable if infrastructure, new buildings and equipment are required, as well as annual running costs for staff and consumables, for example, PCR. The cost for JD surveillance at the import level would be a governmental responsibility, but at the herd level, it would fall on the farmer. The cost of CD surveillance at the hospital level may fall on the healthcare system or the patient. The overall cost of JD surveillance is offset by the huge potential losses in yield and productivity, estimated in New England, Canada, for example, at $15.4M [129]. 

Similarly, MAP surveillance in the food and water industry would have high costs in that it needs to be sensitive and rapid (so as not to hold up distribution of the product), and would be an additional test to those already carried out, for example, for *E. coli*. Given the slow growth of all mycobacteria, including MAP, PCR for IS900 and F57 would be the favoured method, with the possibility of industry standardisation [130]. However, this would have a cost which is not, as yet, quantified. 

## 7. Conclusion and Future Directions

Although the aetiology of CD remains largely unknown, it involves a complex interaction between the genetic, environmental, microbial and immunological factors [131]. MAP may eventually be classed as a zoonotic microbial pathogen. This is the first review to alert the Malaysian sector to the ‘Western experience’ where, as JD increased in prevalence and became endemic, it was later observed that the incidence of CD increased and had a significant association with MAP, the causative agent of JD. Despite the evidence, the precautionary principle has not been applied worldwide, and MAP goes under the surveillance radar in food and water, and in the environment, as no legislation exists that demands this action. 

Bold steps are needed in Malaysia, considering:

- The convincing association between CD and MAP;
- The worldwide MAP-induced endemic of JD;
- The increasing reliance of Malaysia on imported cattle and milk products from areas of high Johne’s prevalence to meet increasing demand;
- Changes in milk preferences in the Malaysian population;
- The low but increasing incidence of CD in Malaysia;
- The proven association of CD and MAP through exposure to milk, dairy products, water, and the environment.

In conclusion, early surveillance programs, in addition to animal control regulations by Malaysian regulators, may stem the clear increase of CD in Malaysia, but at present, it would have to rely on a strategy based on the precautionary principle.

## Figures and Tables

**Table 1:** Incidence of CD and UC in Asia (per 100,000 person-years; modified from [14] (UK data from [5]).

Country	Period	UC	CD
China	2011–2013	1.21	0.34
Hong Kong	2014	1.51	1.46
India	2012–2013	5.40	3.91
Japan	2014	12.2	2.0
Korea	2015	6.58	2.42
Southwest Asia	2011–2013	0.49	0.36
Taiwan	2015	0.95	0.47
UK	2000–2018	15.7	10.2

**Table 2:** Comparison of clinical presentation of CD and UC in the West and East (modified from [19]).

Clinical Presentation	West (W)	East (E)
Age at Diagnosis	Peak 1: 20–39 Peak 2: 60–79	Peak 1: 20–50 Less common after 50
Gender differences	Female > male for CD No difference for UC	Male > female for CD and UC
Phenotype	CD: 70–80% inflammatory UC: no W-E difference	CD: 30% structuring/penetrative symptoms CD More perianal and upper GI UC: no W-E difference
Complications	CD: Same W-E UC: Colectomy > E	CD: Same W-E complications UC: less severe/colectomy less likely
Hospital	W > E High but decreasing	E < W but increasing.

**Table 3:** Genetic mutations that impact the Southeast Asian population (Ethnic groups M-Malay, I-Indian, C-Chinese; W-Western).

Gene	SNP	Gene Product Role and Impact	Susceptible Population	Reference
*NOD2*	SNP5 & JW1	Intracellular sensor for bacteria triggering an immune response. Early onset/stricturing.	I not M	[35]
*ATG16L1*	rs2241880	Autophagy and SNP impair the body’s response to pathogens and contributing to inflammation.	M	[36]
*ATG16L2*	rs1123567	Potential inhibitor or modulator of autophagy. Reduced ATG16L2 expression may lead to increased autophagy. Protective.	M only	[37]
*LINC00824*	rs6651252	Increasing the expression promotes cell growth and proliferation, and chronic inflammation.	M	[38]
*IBD5*	IGR2198a_1 IGR2092a_2	Increased stricturing or penetrating CD.	MI	[39]
*IRF5*	rs3807306 rs10954213	The IRF5 gene has a crucial role in regulating the immune system’s response, leading to an overactive or dysregulated immune system that can cause a hyperactive inflammatory state.		[40,41]
*CXC16*	rs2277680	Pro-inflammatory role in IBD A tenfold increase in CD patients compared with healthy control subjects.	M	[40,42]
*TLR4*	rs4986791	Immune dysregulation.	M	[43]
*DLG5*	Halotype 4136C/A	May result in loss of structure in epithelial cells and increased risk of infection.	M	[44]
*JAK2*	rs10974944	A key role in inflammatory signalling pathways, if unregulated, then inflammation increases.	C	[45]

## Data Availability

Data are contained within the article.

## Abbreviations

The following abbreviations are used in this manuscript:

CD	Crohn’s disease
FISH	Fluorescent *in situ* hybridisation
JAKIM	Jabatan Kemajuan Islam Malaysia, Islamic authorities
JD	Johne’s Disease
DVS	Malaysian Department of Veterinary Services
MAP	*Mycobacterium avium* subspecies *paratuberculosis*
MYR	Malaysian Ringgit (currency)
PCR	Polymerase chain reaction
UC	Ulcerative Colitis
USDA	United States Department of Agriculture
SEA	Southeast Asia
